# Tiling array-CGH for the assessment of genomic similarities among synchronous unilateral and bilateral invasive breast cancer tumor pairs

**DOI:** 10.1186/1472-6890-8-6

**Published:** 2008-07-10

**Authors:** Sara Brommesson, Göran Jönsson, Carina Strand, Dorthe Grabau, Per Malmström, Markus Ringnér, Mårten Fernö, Ingrid Hedenfalk

**Affiliations:** 1 Division of Oncology, Department of Clinical Sciences, Lund University, SE-221 85 Lund, Sweden; 2 CREATE Health Strategic Centre for Clinical Cancer Research, Lund University, SE-221 85 Lund, Sweden; 3 Division of Pathology, Department of Clinical Sciences, Lund University, SE-221 85 Lund, Sweden

## Abstract

**Background:**

Today, no objective criteria exist to differentiate between individual primary tumors and intra- or intermammary dissemination respectively, in patients diagnosed with two or more synchronous breast cancers. To elucidate whether these tumors most likely arise through clonal expansion, or whether they represent individual primary tumors is of tumor biological interest and may have clinical implications. In this respect, high resolution genomic profiling may provide a more reliable approach than conventional histopathological and tumor biological factors.

**Methods:**

32 K tiling microarray-based comparative genomic hybridization (aCGH) was used to explore the genomic similarities among synchronous unilateral and bilateral invasive breast cancer tumor pairs, and was compared with histopathological and tumor biological parameters.

**Results:**

Based on global copy number profiles and unsupervised hierarchical clustering, five of ten (p = 1.9 × 10^-5^) unilateral tumor pairs displayed similar genomic profiles within the pair, while only one of eight bilateral tumor pairs (p = 0.29) displayed pair-wise genomic similarities. DNA index, histological type and presence of vessel invasion correlated with the genomic analyses.

**Conclusion:**

Synchronous unilateral tumor pairs are often genomically similar, while synchronous bilateral tumors most often represent individual primary tumors. However, two independent unilateral primary tumors can develop synchronously and contralateral tumor spread can occur. The presence of an intraductal component is not informative when establishing the independence of two tumors, while vessel invasion, the presence of which was found in clustering tumor pairs but not in tumor pairs that did not cluster together, supports the clustering outcome. Our data suggest that genomically similar unilateral tumor pairs may represent a more aggressive disease that requires the addition of more severe treatment modalities, and underscores the importance of evaluating the clonality of multiple tumors for optimal patient management. In summary, our findings demonstrate the importance of evaluating the properties of both tumors in order to determine the most optimal patient management.

## Background

The incidence of synchronous bilateral breast cancer is low, corresponding to less than 2% of all breast cancer diagnoses [1-3]. The definition of synchronism in this context is, however, ambiguous in the literature, and the time spans used to define breast cancers as synchronous range from one month to two years, making the interpretation of incidence difficult [4-6]. In this study we define synchronously diagnosed breast cancer as the occurrence of two (or more) tumors at primary surgery without a prior history of breast cancer. Synchronous multiple tumors in the same breast are often described as multi-focal or multi-centric (multiple tumors in the same quadrant or in different quadrants of the breast, respectively) and are thought to develop much more often than synchronous bilateral breast tumors. Between one-fourth and half of all patients with breast cancer have been reported to have another carcinoma focus in the same breast in addition to the index tumor [7,8].

When multiple breast tumors are detected synchronously the question arises whether these tumors represent independent primary lesions or whether they are genetically similar. Chaudary *et al*. have set up criteria used to discriminate between independent primary lesions and metastases in synchronous bilateral breast cancer [1]. Bilateral carcinomas are considered independent: 1) if both tumors have *in situ *components, or 2) if the lesions are of different histological types, or 3) if the lesions have different degrees of histological differentiation, and 4) there is no evidence of regional or distant metastases. However, from a clinical perspective, bilateral tumors are most often considered separate tumours due to the limited information obtained from the criteria above. In clinical routine, adjuvant medical therapy is recommended on the basis of tumor biological characteristics of both tumors and adjuvant radiotherapy on type of surgery and number of lymph nodes with metastases. Contralateral metastasis as a first event is very rare, but cannot be completely excluded if the two tumors appear histologically very similar.

To discriminate primary lesions from metastatic spread with multiple carcinomas in the same breast is however more difficult as tumor histology is in most cases inconclusive, although the presence of an intraductal component in each of the lesions suggests independent origins [7]. Other parameters, such as the separation of the lesions by uninvolved breast tissue and the location of the foci in different quadrants of the breast have also been considered indicators of independent primary lesions [7,8]. Adjuvant therapy for multiple unilateral tumors is tailored to the tumor with the worst prognosis, most often the largest tumor, and the presence of lymph node metastases. It was recently shown that unilateral multicentricity was a risk factor for local recurrence in young patients treated with breast conserving surgery [9], emphasizing the clinical significance of this diagnosis.

Studying genetic alterations may provide a more objective assessment when discerning clonal relationships between multiple tumors, or may serve as a complement to existing histopathological evaluations. Methods including X chromosome inactivation analysis, comparisons of allelic imbalance patterns [10-13], or the distribution of *p53 *mutations [14,15] have all been used to address this issue. These studies illustrate that more tumors can be classified as either primary lesions or metastatic spread with the additional analysis of genetic markers, but the concordance with conventional histopathological analysis is low and neither of these methods has given an overall objective assessment of tumor clonality. More recently, karyotyping [8] and metaphase comparative genomic hybridization (CGH) [16,17] have been applied to address the extent of genetic relationship between multiple breast tumors on a genome-wide scale. Using CGH, it was shown that unilateral (multi-focal) tumors usually were more genetically similar to each other than bilateral tumors, indicating that unilateral tumors more often represent intramammary dissemination of a single breast cancer [16]. Metaphase CGH however, only shows copy number gains and losses and not individual gene amplifications or deletions, and the technique is hampered by limited resolution (10–20 Mb). Improvements in resolution and sensitivity of copy number aberrations have recently been made possible through microarray-based CGH (aCGH) [18], which could potentially serve as an important complement to histopathological diagnoses of multiple synchronous breast tumors as recently illustrated by Ghazani and colleagues [19]. To this end, Wa *et al*. used aCGH to study the relationship between two synchronous unilateral breast tumors in a single patient and found the genetic analysis to clearly demonstrate the occurrence of two independent lesions [20].

To our knowledge very little has been published regarding biological characteristics of synchronously diagnosed multiple unilateral and bilateral breast cancer, with the definition of synchronous being no time elapsed between surgical excision of the tumors, *i.e*. without potential interference of adjuvant medical treatment. The present study is the first using high resolution genomic profiling to investigate genomic similarities between tumor pairs from the same patient in a cohort of synchronous unilateral as well as bilateral breast cancers, in combination with histopathological evaluation. The aim of this study was to address the issue of genetic similarity in synchronously diagnosed unilateral (multi-focal or multi-centric) and bilateral breast cancer using tiling resolution aCGH, with the ultimate goal of determining tumor clonality and whether this information can be of clinical significance when making treatment decisions. In addition, the assessment of genomic similarities/dissimilarities was evaluated in relation to conventional histopathological and tumor biological parameters to determine the level of concordance between the genetic evaluation and commonly used histopathological criteria. Our findings demonstrate the importance of evaluating the properties of both tumors in order to determine the most optimal management of the patient.

## Methods

### Patients and tumors

Patients diagnosed with synchronous unilateral or bilateral breast tumors between 1987 and 2006 at Lund University Hospital from whom sufficient material from both tumors was available in the Southern Sweden Breast Cancer Group's tissue bank at the Departments of Oncology and Pathology, Lund University Hospital, were included in the study. In addition, formalin-fixed, paraffin-embedded (FFPE) tumor pairs from a small number of patients from the same institution were included to increase sample numbers. Within all pairs, both tumors were either fresh frozen (n = 15 pairs) or FFPE (n = 3 pairs). We defined synchronously diagnosed breast cancer tumors as having the same date of surgical excision. A total of 33 tumors, representing tumor pairs from 16 patients, were analyzed. One of the 16 patients had three synchronous tumors (two in the left breast and one in the right), which for the subsequent analyses resulted in one unilateral tumor pair and two bilateral tumor pairs from this patient. Unilateral breast cancer was defined as two discrete tumors in the same breast separated by at least 1 cm of normal tissue. Altogether, ten unilateral tumor pairs and eight bilateral tumor pairs were included in the analyses. Two lymph nodes from patients in the cohort were included to obtain a measurement of genomic similarity (FFPE material). Only one patient (patient 15) received pre-operative treatment; radiotherapy and neo-adjuvant chemotherapy (ACFUMx6; adriamycin, cyclophosphamide, 5-fluorouracil, metothrexate). A family history of breast cancer was only known for one patient (patient 2). The study was approved by the Lund University ethics committee.

### Histopathology and immunohistochemistry

All samples were re-evaluated by a pathologist (DG) to confirm histological type and grade (Nottingham histological grade, NHG) [21] presence or absence of an intraductal component, vessel invasion (based on H&E stained sections), tumor size and nodal status, according to routine practice. Estrogen receptor (ER) and progesterone receptor (PgR) status were determined by enzyme immunoassay (fresh frozen tissue, according to Abbott Laboratories (Chicago, IL)), or by immunohistochemical assay (FFPE tissue), and routine DNA index (flow cytometric DNA analysis) was assessed prospectively according to standard procedures as previously described [22-24].

### DNA isolation

Genomic DNA was isolated from frozen and FFPE tissue using proteinase K followed by the Wizard Genomic DNA extraction kit and phenol chloroform purification according to the manufacturer's protocols (Promega Corporation, Madison, WI), and DNA concentrations were measured using the NanoDrop Spectrophotometer (NanoDrop Technologies, Wilmington, DE). To test the DNA integrity from FFPE tissue, an amplification according to a random amplified polymorphic protocol (Amersham Bioscience, Piscataway, NJ) was performed; DNA quality was then tested using the Agilent DNA 1000 Kit with the Agilent BioAnalyzer 2100 system (Agilent Technologies, Santa Clara, CA). Visual inspection of the electropherograms was performed, and samples with unsatisfactory DNA quality were excluded.

### Array comparative genomic hybridization

Microarrays with complete tiling genomic coverage were produced from the 32 K BAC clone library (CHORI BACPAC Resources [25]) at the Swegene DNA Microarray Resource Centre, Department of Oncology, Lund University, Sweden. Mapping data for each clone was based on the UCSC May 2004 assembly (hg17). Arrays were constructed as described elsewhere [26]. Three μg of genomic DNA from samples and two μg of male commercial reference DNA (Promega Corporation, Madison, WI) was labeled and hybridized, and arrays were scanned according to previously published protocols [27,28]. To account for differences in dye incorporation with DNA from FFPE tissue, dye swap experiments were performed for these samples.

### Data and image analysis

Gene Pix Pro 4.0 (Axon Instruments Inc., Union City, CA) was used to identify individual spots on scanned arrays and the data was processed and analyzed using BioArray Software Environment BASE [29]. The Cy3 and Cy5 intensities were background-corrected by calculating the median-feature and median-local background intensities provided in the quantified data matrix. Within each of the arrays, ratios of intensity for individual probes were calculated as background corrected intensity of the sample, divided by background corrected intensity of the reference sample. Log(2)ratios were normalized and corrected for intensity-based location adjustment [30]. The X and Y chromosome BAC clones were excluded during the normalization. To reduce experimental noise over the chromosomal profile a moving average of 400,000 base pairs was applied. A BASE implementation of CGH Plotter was used to determine deletion/amplicon boundaries [31]. Further evaluation of the genetic similarity/dissimilarity between tumors from the same patient was performed by unsupervised hierarchical clustering of segmented CGH data. Clustering based on global genomic profiles was performed with average linkage and Pearson correlation.

### Statistical analysis

A random permutation test was performed to validate the significance of the number of unilateral or bilateral tumor pairs that exhibited a high degree of genomic similarity. The sample labels of the arrays were randomly permuted, whereupon the number of unilateral and bilateral tumor pairs, respectively, that clustered together was calculated. This procedure was performed 10^6 ^times. Using these permutations, the actual numbers of unilateral and bilateral tumor pairs that clustered together were assigned p-values corresponding to the probability to obtain as many clustered pairs or better under this null hypothesis of genomic profiles randomly associated with sample labels.

## Results

### Array comparative genomic hybridization

Several non-random chromosomal aberrations were found in the tumor cohort. The most commonly observed gains were located on chromosomes 16p (50%), 8q (45%), 1q (40%) and 10p (25%), whereas the most common losses were found on chromosomes 16q (45%), 21 (30%), 8p (30%) and 13 (25%). A summary of the genomic aberrations of each individual tumor is provided in Additional File 1 (Summary of genomic aberrations in uni- and bilateral breast cancer tumor pairs). Due to the preponderance of tumors with a high histological grade, the prevalence of chromosomal aberrations was complex in many samples.

**Figure 1:**
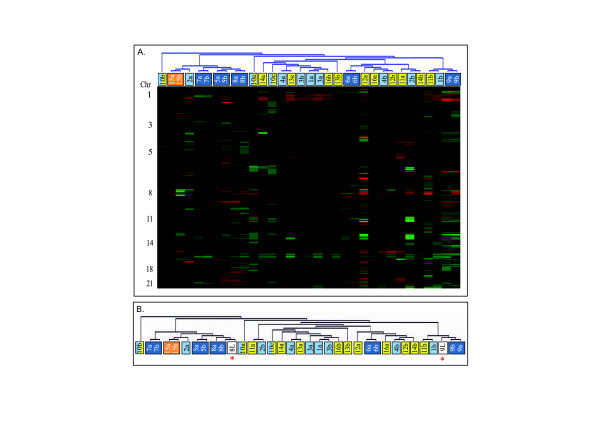
**Unsupervised hierarchical clustering analysis of genomic similarities between synchronously diagnosed unilateral and bilateral tumor pairs from 16 breast cancer patients**. **A**. Dark blue boxes represent unilateral tumor pairs from the same patient displaying genomic similarities. Light blue boxes represent genomically dissimilar unilateral tumor pairs. The orange box represents a bilateral tumor pair displaying genomic similarities, and yellow boxes represent genomically dissimilar bilateral tumor pairs. Patient number 10 was diagnosed with three synchronous tumors, resulting in one unilateral tumor pair and two bilateral pairs (light blue and yellow boxes). **B**. The addition of two matched lymph nodes (white boxes marked by a red star) to the unsupervised hierarchical clustering analysis resulted in the two lymph nodes clustering with the tumor pair from the corresponding patient.

Unsupervised hierarchical clustering analysis was used to identify tumor pairs showing similar genome-wide DNA copy number profiles (Figure 1A). The connection of two tumor samples from the same patient on short branches in the dendrogram indicates that these genetic profiles are more similar to each other on a genome-wide basis than to any other tumor sample in the cohort. To validate this approach we included two matched lymph nodes to the analysis. This resulted in a close clustering of the lymph nodes with the tumors from the respective patients (Figure 1B), confirming their respective mutual genomic origins. The source of the DNA (*i.e*. from fresh frozen or FFPE tissue; see Table 1) did not seem to affect the clustering outcome, as tumor pairs from either source were found among those that clustered as well as among the non-clustering pairs. Such similarity measures have been found to be useful for aCGH profiles [32,33], in particular for tumor specimens as they often contain a varying degree of stromal contamination. We were in the present investigation only interested in identifying tumor pairs that appeared as pairs in the hierarchical clustering analysis. Importantly, this approach is independent of the choice of linkage method used.

**Table 1:** Patient and tumor characteristics.

**Tumor**	**Distance between tumors (cm)**	**Grade**	**ER status**	**PgR status**	**DNA index**	**Intraductal component**	**Node status**	**Vessel invasion**
**Unilateral tumor pairs that do not cluster**								
1a	6	1	pos	pos	1.61	yes	neg	no
1b§		2	pos	pos	1.00	no		
2a	5	3	neg	neg	1.80	yes	neg	no
2b		2	pos	neg	1.58	yes		
3a	4	1	pos	pos	1.08	yes	pos	no
3b		1	pos	pos	1.00	yes		
4a§	n/a	2	pos	pos	1.10	yes	pos	no
4b		2	pos	pos	1.16	no		
10a	n/a	2	pos	pos	1.00	yes	pos	no
10b		2	pos	pos	1.00	yes		
**Unilateral tumor pairs that cluster**								
5a	2	3	neg	neg	1.45	yes	pos	yes
5b		3	neg	neg	1.56	yes		
6a	1	3	pos	pos	1.70	yes	pos	no
6b		3	pos	pos	1.73	yes		
7aγ	3	3	neg	pos	1.98	yes	neg	yes
7bγ		3	pos	pos	1.81	yes		
8aγ	1	3	neg	neg	2.50	yes	pos	yes
8bγ		3	neg	neg	2.28	no		
9aγ	8	3	pos	pos	1.63	yes	pos	yes
9bγ		3	pos	pos	1.73	yes		

**Bilateral tumor pairs that do not cluster**								
10a	-	2	pos	pos	1.00	yes	pos	no
10c		2	pos	pos	1.00	yes	neg	no
10b		2	pos	pos	1.00	yes	pos	no
10c		2	pos	pos	1.00	yes	neg	no
11a	-	1	pos	neg	2.83	yes	neg	no
11b		3	pos	neg	1.00	no	pos	no
12a	-	3	pos	pos	2.73	no	neg	no
12b		1	pos	pos	1.89	no	neg	no
13a	-	2	pos	pos	1.09	yes	pos	no
13b		2	pos	pos	1.00	yes	pos	no
14a	-	2	pos	pos	1.75	yes	pos	no
14b		2	pos	pos	1.76	yes	pos	yes
16a	-	1	pos	pos	2.19	yes	pos	no
16b		2	neg	neg	1.00	yes	n/a	no
**Bilateral tumor pairs that cluster**								
15a	-	3	neg	neg	1.94	yes	pos	no
15b		3	pos	pos	2.01	yes	pos	no

Patients are numbered consecutively and tumor pairs are denoted a and b, respectively. All tumors, except where otherwise indicated, were of ductal type. Unless otherwise indicated, all samples were from fresh frozen tissue.

§ lobular type

γ FFPE

**Figure 2:**
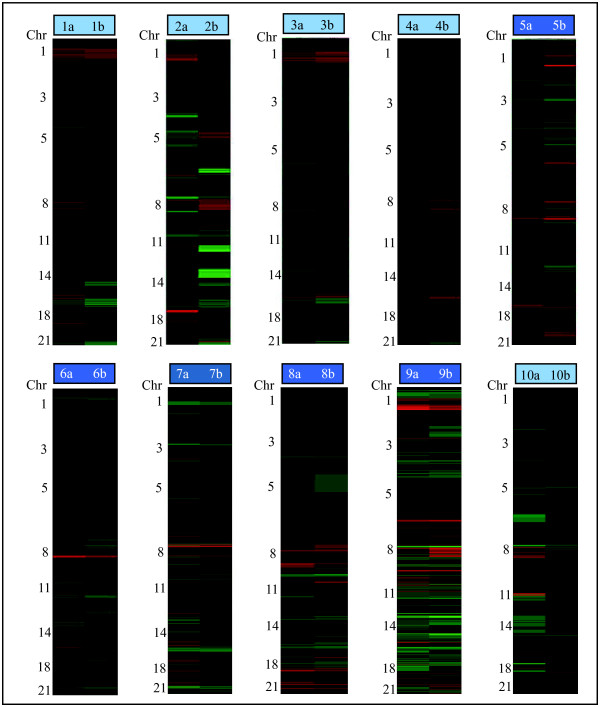
**Heatmaps of chromosomal aberrations in ten synchronously diagnosed unilateral breast tumor pairs**. Gains/amplifications are shown in red and losses/deletions in green. Dark blue boxes indicate genomically similar tumor pairs and light blue boxes indicate genomically dissimilar tumor pairs.

Of the ten patients with two unilateral breast tumors (n = 7 frozen, n = 3 FFPE) a high degree of genomic similarity was suggested within the tumor pairs of five patients (n = 2 frozen, n = 3 FFPE; Figure 1A; dark blue boxes). This is significantly more than expected by random permutation of the sample labels (p = 1.9 × 10^-5^). Notably, despite numerous and complex chromosomal aberrations in both tumors within these pairs, they clustered together. Discrepancies in aberration amplitudes between the tumors in the clustering pairs was, however, seen in some cases, *e.g*. chromosomes 11q and 17 in tumor pair 6, and chromosomes 5 and 8q in tumor pair 8 (Figure 2); but since the assessment of genomic similarity is based on patterns of aberrations, the amplitude does not affect the outcome of the clustering analysis.

**Figure 3:**
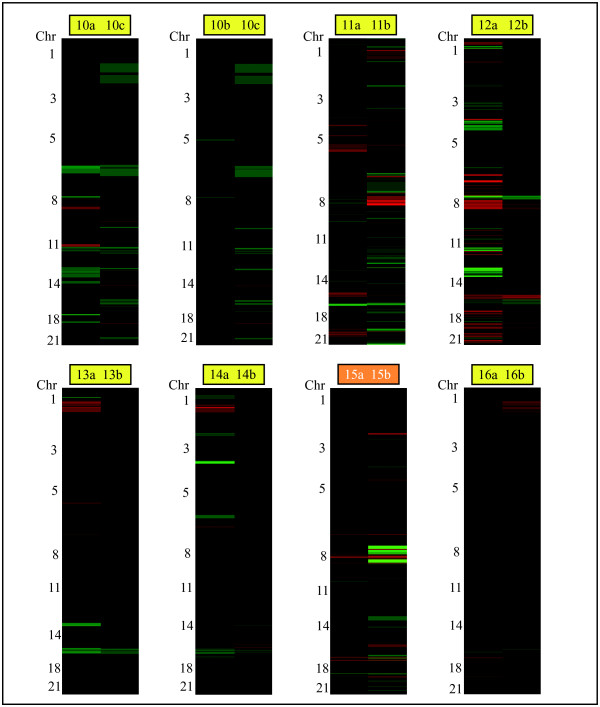
**Heatmaps of chromosomal aberrations in eight synchronously diagnosed bilateral breast tumor pairs**. Gains/amplifications are shown in red and losses/deletions in green. The orange box indicates the genomically similar tumor pair, and the yellow boxes indicate genomically dissimilar tumor pairs.

Among the bilateral tumor pairs, similarities in the genome wide copy number profiles were only apparent in one of eight tumor pairs (Figure 1A; orange box), which is not significant compared to random expectations (p = 0.29). This tumor pair displayed a multitude of complex chromosomal aberrations as well as variations in amplitude (Figure 3; tumor pair 15).

### Unilateral tumor pairs

Two of the five unilateral tumor pairs that did not cluster together (of the ten unilateral pairs) demonstrated a high degree of difference in chromosomal imbalances, suggesting genetic heterogeneity, and hence that two synchronously diagnosed unilateral breast tumors can have different origins. An example of a tumor pair of possibly different origins is tumor pair 2 where one tumor (2b) harbored an *ERBB2 *amplification, whereas the other (2a) did not. Also tumor pair 4 displayed a number of major differences in copy number profiles (gain of chromosome 1 in tumor 4a, and chromosome 8 in tumor 4b, loss of chromosome 9p in tumor 4a and chromosome 22 in tumor 4b; see Figure 2), indicating unilateral tumor heterogeneity.

For the remaining three pairs in the group that did not cluster together, the tumors exhibited few chromosomal aberrations; moreover these imbalances are commonly found in breast tumors (*e.g*. gain of chromosome 1q, loss of chromosomes 8 and 16q). Even small differences may nonetheless have a profound effect on the clustering outcome; *e.g*. despite only differing on chromosomes 5 and 8, the tumors within tumor pair 1 did not cluster together (Figure 2). In addition, the slight differences between the tumors on chromosomes 6, 8 and 22 in tumor pair 3 resulted in these tumors not clustering as a pair (Figure 2). The tumors from these two patients were all histological grade 1, except tumor 1a which was histological grade 2. Patient number 10 was diagnosed with three synchronous breast tumors, two in the left breast and one in the right breast, thereby giving rise to one unilateral tumor pair (10a and b; Figure 2) and two bilateral tumor pairs (10a and c, and 10b and c; Figure 3) for comparison. Neither of these tumors clustered together, suggesting that the three tumors all developed from different origins.

Of the five unilateral tumor pairs that exhibited similar genomic profiles within the pair (of the ten unilateral pairs), both tumors in all of the pairs were of the same histological grade (grade 3; see Table 1). In addition, the range in DNA indices showed only minor variations for tumors that clustered together, while tumors that did not cluster together showed a DNA index range with greater degrees of variation (range: 2–10% *vs*. 0–61%). For two of the pairs that did not cluster together, the evaluation of histological type differed between the tumors, one tumor being ductal type and the other lobular type in these pairs. All other tumors in the cohort were of ductal type.

Vessel invasion was observed in four of the ten unilateral tumor pairs, all of which clustered together as pairs in the genomic analysis (Table 1). Four of the five tumor pairs in the group that exhibited genomic similarities had an intraductal component in both tumors, while three of five tumor pairs in the group that did not cluster together had an intraductal component in both tumors (Table 1), suggesting that the presence of an intraductal component in the unilateral tumor pairs that cluster together represents an intraductal growth phase rather than pre-invasive *in situ *cancer. Tumor size, hormone receptor status, distance between tumors and lymph node status were inconclusive or did not correlate with the results of the genomic similarity analysis.

### Bilateral tumor pairs

Only one of the eight bilateral pairs exhibited genomic similarities in the clustering analysis; both tumors in this pair contained an intraductal component and both were histological grade 3. The DNA indices for these two tumors were similar (1.94 and 2.01), and this patient had positive lymph nodes on both right and left sides. For the seven bilateral pairs that did not cluster together, four were concordant with respect to histological grade within the pair while three were not (Table 1). Three of the seven pairs had a greater variation in DNA index between the tumors than the clustering pairs (range 18–183%), whereas the remaining four pairs only had a minor or no variation in DNA index (range 0–9%). Five of the seven tumor pairs that did not cluster had an intraductal component in both tumors. The sixth patient had one tumor that contained an intraductal component while the other tumor did not, and in the seventh patient neither tumor had an intraductal component; nevertheless this tumor pair did not cluster together. As was the case for unilateral tumors, bilateral tumor pairs that did not cluster together (with the exception of tumor pair 14) had no vessel invasion. Tumor size, hormone receptor status and lymph node status were inconclusive or did not correlate with the results of the genomic similarity analysis.

## Discussion

CGH has greatly facilitated the detection of gains and losses in DNA copy number, and is especially useful when elucidating patterns of genomic alterations in tumors. Since the extent of genomic imbalances can be determined with greater mapping precision due to the high resolution and large number of data points [19], aCGH in particular may be of clinical value in differentiating new primary tumors from recurrent lesions and genomic analysis can help reveal the relationship between multiple tumors. Moreover, this may have significant implications for selection of optimal adjuvant treatment. Today, differences in tumor histopathology are used to distinguish between disseminated disease and the occurrence of multiple synchronous primary tumors. Recent reports, however, demonstrate that histopathological evaluation fails to provide unambiguous evidence for tumor origin; hence it has been proposed that genetic analyses are more reliable in this context [9,14,16]. In the present study we elucidated genomic alterations in synchronously diagnosed unilateral and bilateral breast tumor pairs using 32 K tiling BAC aCGH. The analysis of tumor pairs from 10 patients diagnosed with synchronous unilateral breast cancer by unsupervised hierarchical clustering resulted in tumors from five patients clustering together as pairs, suggesting a genomic similarity and a possible common origin of the two tumors in these patients. These tumor pairs clustered together despite complex chromosomal copy number aberrations. In line with our findings, previous studies based on genetic as well as histopathological evaluations have concluded that a large fraction of unilateral breast tumors arise through intramammary dissemination of a single breast cancer, *i.e*. suggesting that most unilateral tumors develop from the same clonal origin [11,16,34-36]. More recently, similar results have been obtained by analyzing unilateral breast tumors using metaphase CGH and unsupervised hierarchical clustering; one such study revealed that paired tumors from three of four unilateral breast cancer patients demonstrated a clonal relatedness based on copy number profiles [16]. Alternatively, it is possible that the genomic similarities observed in our study could be caused by a 'field effect' whereby *e.g*. a common environmental exposure could cause the development of genomically similar, but independent, tumors. However, this appears less likely, as such an environmental effect would be expected to affect the similarity also among bilateral cases, which was not the case. In our study, an absence of genomic similarities was found in five of ten synchronous unilateral tumor pairs, *i.e*. the two tumors from the same patient did not cluster together. Two of these tumor pairs displayed highly discordant chromosomal imbalances, emphasizing the possibility that the tumors have developed independently. Alternatively, intra-tumor heterogeneity may explain these differences, as recently suggested [17]. Based on these findings we conclude that synchronous unilateral tumors often are genetically similar, indicating that they develop from a single clonal origin, but multiple individual primary carcinomas might also develop synchronously in the same breast, confirming earlier studies [8,16,19,36].

Only one of eight synchronously diagnosed bilateral breast tumor pairs displayed genomic similarities in the clustering analysis. Based on clinical as well as histopathological variables, this patient most likely presented with generalized disease, further underscoring the clonal relatedness between the tumors. This patient also received radiation and chemotherapy prior to surgery, which may potentially have affected the genomic analyses; however this does not seem to be the case because no pathological response to the treatment was observed. Histopathological parameters that support the notion of contralateral spread include positive lymph nodes on both sides, the same histological grade (3) and type (ductal carcinoma), all of which have been suggested as evidence for metastatic disease [1,3]. In contrast, both tumors displayed intraductal carcinoma, one of the criteria used to determine lesions of independent origin in bilateral breast cancer [1].

Earlier studies of bilateral breast cancer have demonstrated a failure to provide unambiguous evidence for the distinction between independent tumors and contralateral spread based on histopathological parameters [10,14,15]. Of the tumor biological and histopathological parameters investigated in our study, DNA index and histological type correlated with the genetically based clustering outcome. Specifically, all six tumor pairs that displayed genomic similarities within the pair also had highly similar DNA indices, and both tumors within the pair were of the same histological type in these cases. Among the tumors that did not illustrate pair-wise genomic similarities in the clustering analysis, two tumor pairs had different histological types (tumors 1b and 4a were of lobular type and tumors 1a and 4b were of ductal type), and seven of ten tumor pairs displayed large differences in DNA index within the tumor pairs. Tumor size, hormone receptor status, distance between tumors (for unilateral tumors) and lymph node status were inconclusive or did not correlate with the results of the genomic analysis. Nevertheless, one study has demonstrated that the distance between the tumors might be of significance when discriminating between independent tumors and intramammary dissemination in multi-focal or multi-centric unilateral tumors; the study was however very small and no significance was reached [8].

Since tumor histology (type and grade) is inconclusive in most cases of multiple tumors in the same breast, it is difficult to discriminate between the development of multiple primary lesions and lesions developed from the same tumor origin. Based on the notion that intraductal cancer constitutes a pre-invasive phase in breast cancer progression, it has been suggested that the presence of intraductal components in each of the lesions indicates independent origins [7,8]. However, more recent studies suggest that the occurrence of an intraductal component is of little help in distinguishing multiple independent tumors from intramammary dissemination [16,35,36]. The fact that intraductal carcinoma occurred at the same frequency in clustered pairs as in non-clustered pairs in our study (four of five *vs*. three of five), together with the observation that the grade of the intraductal component was similar to that of the corresponding invasive component, suggests that the intraductal component represents an intraductal growth phase rather than pre-invasive *in situ *cancer and confirms the view put forward in the latter studies. By histopathological examination, it is very difficult to distinguish between pre-invasive carcinoma *in situ *and intraductal spreading of invasive components. However, in our study, the unilateral tumor pairs that clustered together (four of five) demonstrated vessel invasion while none of the pairs that did not cluster displayed this feature, suggesting a common origin of the tumors that clustered together. This stresses the importance of determining the ability of a tumor to spread and invade surrounding tissue by distinguishing between a pre-invasive *in situ *component and intraductal spreading by evaluating vessel invasion in conjunction with the intraductal component.

Even if a majority of unilateral breast tumors arise through intramammary dissemination of a single breast cancer a significant number of synchronous unilateral tumors could, according to our study, have developed from independent origins. This might be important to consider when evaluating the clinical outcome of the patient. Our findings demonstrate the importance of evaluating the properties of both tumors in order to determine the most optimal treatment for the patient. The possibility of synchronous unilateral tumors representing individual clones could have implications for the clinical outcome of the patient if both tumors are not accurately characterized. Using a PCR-based approach, it was recently shown that clonally related ipsilateral recurrences were more frequently of higher histological grade and developed sooner after initial treatment than clonally distinct ipsilateral recurrences [9], and it has also been reported that women with clonally related ipsilateral recurrences had poorer outcomes than patients with unrelated tumor pairs [37]. In the present study, all the unilateral tumor pairs that clustered together were histological grade 3, while the non-clustering tumor pairs were low to moderate histological grade (see Table 1). Molecular approaches may hence provide a reliable means of identifying patients who may benefit from more aggressive systemic treatment, *i.e*. whose tumors are clonally related and more likely to be associated with metastatic disease. Of interest, a recent study of 22 pairs of ipsilateral breast cancers revealed a statistical difference in metastasis-free survival between new primary tumors and recurrences as defined by a 'partial identity score' based on DNA breakpoint information, suggesting that genomic analyses could outperform clinical and histopathological characteristics in terms of prediction of prognosis [38], and supporting our conclusions. The importance of correctly characterizing the individual synchronous tumors is also apparent in bilateral breast cancer. Even if rare, bilateral metastatic spread seems to occur, and since metastatic disease is known to result in a worse prognosis it is important to consider the occurrence of metastatic disease in these cases by analyzing both tumors. Our results clearly demonstrate that through the evaluation of both tumors in the pair by genome-wide aCGH and hierarchical clustering analysis one might obtain objective information regarding whether the tumors are independent lesions or disseminated disease.

## Conclusion

In conclusion, we report that by using tiling aCGH we can obtain an objective measure of genetic similarity in synchronously diagnosed multiple breast tumors. Unilateral tumors with highly similar genomic profiles are thought to share a common origin, while the development of separate primary unilateral breast tumors also occurs based on the genomic dissimilarity within the pairs in these cases. Conversely, synchronous bilateral tumors are most likely individual primary tumors, but contralateral dissemination does occur. Moreover, we found that standard pathological evaluation does not allow a firm determination of clonal relationships among synchronously diagnosed breast tumors, and there was a high level of discordance between conventional parameters and the genetic analysis. A larger study is needed to definitely confirm or refute correlations between conventional histopathological criteria and aCGH-based genomic analyses. We propose that unsupervised hierarchical clustering of aCGH profiles can be used as an objective method to complement standard pathological diagnoses when attempting to reveal clonal relationships among synchronous breast tumors.

## Abbreviations

aCGH: microarray-based comparative genomic hybridization; FFPE: formalin-fixed paraffin-embedded; NHG: Nottingham histological grade; ER: estrogen receptor; PgR: progesterone receptor.

## Competing interests

The authors declare that they have no competing interests.

## Authors' contributions

SB carried out the aCGH experiments with the assistance of GJ and CS and drafted the manuscript. DG performed the pathological review, PM reviewed patient charts and MR performed the statistical analyses. MF and IH conceived of, designed and coordinated the study, and helped draft the manuscript. All authors contributed to the interpretation of the results and approved the final manuscript.

## Pre-publication history

The pre-publication history for this paper can be accessed here:



## Supplementary Material

Additional File 1 **Summary of genomic aberrations in uni- and bilateral breast cancer tumor pairs**. Complete ternary raw data of genomic aberrations in uni- and bilateral tumor pairs from 16 patients included in the study. This tab-delimited text file (3.5 MB) can be viewed with *e.g*. Microsoft Excel. Click here for file
